# What Is the Spatial Extent of a *Bemisia tabaci* Population?

**DOI:** 10.3390/insects11110813

**Published:** 2020-11-18

**Authors:** Michael S. Crossley, William E. Snyder

**Affiliations:** Department of Entomology, University of Georgia, Athens, GA 30602, USA; wesnyder@uga.edu

**Keywords:** fixation index, genetic differentiation, genetic diversity, heterozygote deficit, isolation by distance, microsatellites, population genetics, sweet potato whitefly

## Abstract

**Simple Summary:**

Pest management can be greatly enhanced by basic knowledge about pest dispersal patterns in agroecosystems, which for insects often relies on comparisons of genetic variation among populations. The globally invasive sweet potato whitefly *Bemisia tabaci* is one such pest for which a large body of research has examined patterns of genetic variation. We review this literature to address the question: What spatial scales define *B. tabaci* populations? These studies are global in coverage and draw from a variety of genetic marker types. We found that genetic differentiation among populations is typically low, and that patterns of genetic diversity suggest that groups of migrating whiteflies from divergent populations are typically being sampled together. Overall, these results suggest that there is high ongoing gene flow over large spatial extents, but recent invasion by most populations could obscure genetic markers’ ability to detect geographic isolation. Genome-wide data collected across finer spatial and temporal scales hold great promise to clarify the spatial extent of a *B. tabaci* population, and could reveal whether insecticide rotations can be tailored to specific commodities or if coordination across commodities and regions linked by *B. tabaci* gene flow is justified.

**Abstract:**

Effective pest management depends on basic knowledge about insect dispersal patterns and gene flow in agroecosystems. The globally invasive sweet potato whitefly *Bemisia tabaci* (Gennadius) (Hemiptera: Aleyrodidae) is considered a weak flier whose life history nonetheless predisposes it to frequent dispersal, but the scale over which populations exchange migrants, and should therefore be managed, is uncertain. In this review, we synthesize the emergent literature on *B. tabaci* population genetics to address the question: What spatial scales define *B. tabaci* populations? We find that within-species genetic differentiation among sites is often low, and evidence of population structuring by host plant or geography is rare. Heterozygote deficits prevail among populations, indicating that migrants from divergent populations are frequently sampled together. Overall, these results suggest that there is high ongoing gene flow over large spatial extents. However, genetic homogeneity typical of recently invading populations could obscure power to detect real isolation among populations. Genome-wide data collected systematically across space and time could distinguish signatures of invasion history from those of ongoing gene flow. Characterizing the spatial extent of *B. tabaci* populations could reveal whether insecticide rotations can be tailored to specific commodities or if coordination across linked commodities and regions is justified.

## 1. Introduction

Basic knowledge about insect dispersal patterns in landscapes is foundational to effective agricultural pest management. This is because insects must regularly disperse to colonize crops, and knowing the pathways, timing, and severity of these dispersal events can reveal where, when, and how intensely to apply control measures. Such knowledge can be difficult to obtain; however, for widely dispersing insects whose populations cannot readily be tracked across agricultural landscapes [1,2,3]. In these systems, population genetics approaches, which use observations of genetic diversity and differentiation among populations based on molecular markers to infer evolutionary processes acting on populations, can delineate which host plants are being attacked [4], how far and regularly pests are moving across landscapes [5,6], and whether insecticide resistance (or other traits allowing adaptation to new management techniques) tend to rapidly spread among and across populations [7]. One such agricultural pest for which a substantial body of literature has emerged, using population genetics approaches, is the sweet potato whitefly *Bemisia tabaci* (Gennadius) (Hemiptera: Aleyrodidae) cryptic species complex. The insect’s small body size (0.80−0.95 mm [8]) and wide host range (at least 500 plant species [9]) make *B. tabaci* difficult to physically mark and track as they move among fields and crop species, though movements among crops can be inferred from extensive monitoring efforts that record sequential abundance peaks in one crop and then another [10,11]. Further complicating matters, *B. tabaci* populations often include several “cryptic species” (sometimes called biotypes) that are genetically distinct but morphologically identical [9,12], and whiteflies appear to be readily moved among regions, countries, and even continents when horticultural plants and infested crops are traded [13,14,15,16,17]. All of these traits further complicate surveillance efforts that rely purely on physically tracking whitefly abundance. Rather, it often is necessary to look at underlying genetic variation to infer population boundaries, and gain some indirect indication of how, when, and where they are moving from one site and/or crop to another [18].

Two species within the *B. tabaci* complex have emerged over the last four decades as major economic pests of field and horticultural crops worldwide, named for their geographic origins: Middle East-Asia Minor 1 (hereafter, MEAM 1) and Mediterranean (hereafter, MED) [12]. Though morphologically indistinguishable [9,12], these species diverged ~12 mya [19] and are distinct in economically significant ways. MEAM 1 is characterized by relatively high fecundity [20], mating interference [21], virus transmission [22], vector-virus mutualisms [23,24], and association with physiological disorders in some host plants [25,26]. MED is often associated with greenhouse plant production [13,27] and seems more prone to evolve resistance to insecticides [28,29]. Invading populations of MEAM 1 and MED tend to rapidly sweep through agricultural regions, displacing resident *B. tabaci* species [27,30,31,32,33]. Though considered a weak flier [8], *B. tabaci* is capable of sustained flight over long distances [34], even producing what might be considered a migratory form under some circumstances [35,36]. Yet, a large proportion of dispersing *B. tabaci* will typically fly close to the ground (<10 cm [37]) and settle on nearby host plants [35,38]. The extent to which long-distance migration and the accumulation of short distance movements over many generations contribute to population connectivity over large spatial extents is uncertain, but the genetic consequences of such movements can be examined using a population genetics framework. Understanding the spatial extent of *B. tabaci* populations could potentially aid in designing insecticide rotations that account for resistance in genetically connected areas and targeting efforts to mitigate virus spread by identifying high risk fields.

**Figure 1:**
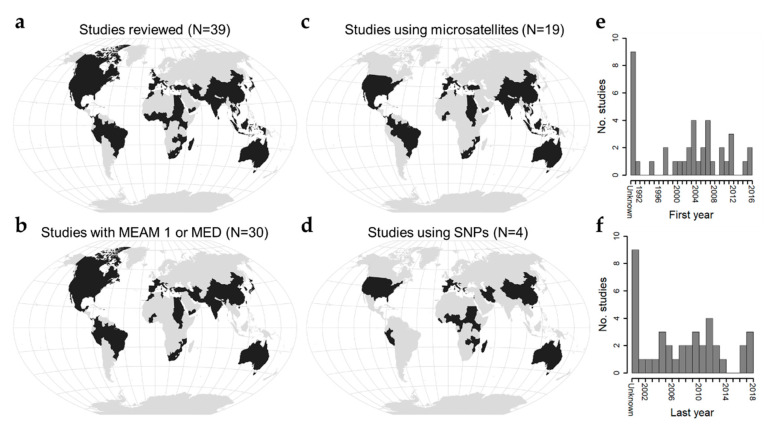
Geographic and temporal scope of literature reviewed. (**a**) Countries where *Bemisia tabaci* populations were sampled in the reviewed literature (number of studies = 39). (**b**) Countries where Middle East-Asia Minor 1 (MEAM 1) and/or Mediterranean (MED) cryptic species were sampled (number of studies = 30). (**c**) Countries where microsatellite data were used to study *B. tabaci* genetic variation (number of studies = 19). (**d**) Countries where single nucleotide polymorphism data were used to study *B. tabaci* genetic variation (number of studies = 4). Maps were drawn using the Winkel Tripel projection. (**e**) Histogram depicting the first year when sampling was reported (range 1991–2016). Studies that did not report sampling dates appear on the left and are labeled as “Unknown”. (**f**) Histogram depicting the last year when sampling was reported in studies (range 2002–2018).

In this review, we synthesize the literature on *B. tabaci* population genetics to address the question: What spatial scales define *B. tabaci* populations? These studies are global in coverage, focus largely on populations of MEAM 1 and MED, and span collections from 1991−2018 (Figure 1, Table S1). Studies leverage a variety of genetic marker types [27,39,40,41,42,43,44,45,46,47,48,49,50,51,52,53], but, while the majority use microsatellites [13,16,17,54,55,56,57,58,59,60,61,62,63,64,65,66,67,68], use of single nucleotide polymorphisms is beginning to become more common [15,69,70,71] (Figure 1). We organize our synthesis around insights gained from three common marker types (cytochrome oxidase I, microsatellites, single nucleotide polymorphisms) and associated measures of genetic diversity and differentiation, interpreting emergent patterns in light of potential underlying ecological and evolutionary processes. In conclusion we suggest three guiding questions for future research that could build upon these findings to further understanding of the relevant spatial scales over which *B. tabaci* should be managed.

## 2. Cytochrome Oxidase I

Sequence data from the mitochondrial gene cytochrome oxidase I (COI) has been used to delineate cryptic species [12], detect invasions and displacement of indigenous species [27,41,42,45,49], describe population size changes [45], and characterize levels of genetic diversity within species [13,41,44,53]. Perhaps unsurprisingly, COI-based studies typically report low levels of genetic diversity among invasive populations of MEAM 1 and MED, with MEAM 1 populations often comprising a single haplotype across entire regions [13,27,56,72]. This is to be expected because mutations in COI do not accumulate quickly enough to enable differentiation of populations within species on ecological time scales [19,73]. In addition, asymmetric gene flow due to cytoplasmic-incompatibility caused by *Wolbachia* can constrain gene flow among uninfected and infected *B. tabaci* lineages, further limiting recovery of genetic diversity among invasive populations [67,74]. While clearly an important first step toward describing genetic differences among invasive lineages, COI sequence comparisons are not well suited for quantification of genetic diversity and differentiation at the smaller spatial and temporal scales that are relevant to processes acting on populations. For this, we turn next to microsatellites.

## 3. Microsatellites

Microsatellites are highly polymorphic, nuclear markers that can parse levels of genetic diversity and differentiation among populations at relatively fine scales [18]. Unlike studies based on COI, microsatellite-based studies frequently report high levels of genetic diversity within invasive *B. tabaci* populations [43,46,55,56,64], though sometimes this is conflated with variation among cryptic species [46] or is indirectly inferred [43]. Some studies report increasing [65] or decreasing [13,45,58] genetic diversity over time, invoking extinction/recolonization dynamics or ongoing population expansion. Relevant to the question of what spatial scales best define *B. tabaci* populations, we examine four themes among *B. tabaci* microsatellite datasets that can shed light on the extent of gene flow among *B. tabaci* populations.

### 3.1. Low Genetic Differentiation

**Figure 2:**
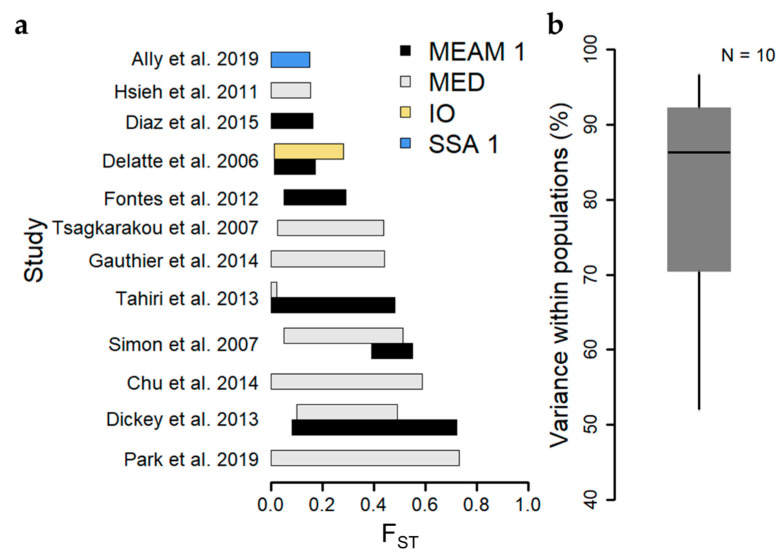
Summary of genetic differentiation among populations within cryptic species among microsatellite-based studies. (**a**) Barplots depict the range of pairwise fixation index (F_ST_) reported among populations within cryptic species and studies. “MEAM 1” stands for Middle East-Asia Minor 1; “MED” stands for Mediterranean; “IO” stands for Indian Ocean; “SSA 1” stands for Sub-Saharan Africa 1. (**b**) Boxplot depicts the distribution of reported values of the percentage of genetic variance partitioned within populations. Boxplots depict the median (thick line), 25th and 75th percentiles (box edges), and 95th percentiles (whiskers).

Genetic differentiation arises when populations are isolated in space over time, due to the random generation and loss of genetic variation by mutation and genetic drift, respectively, and the action of natural selection in their respective environments. Importantly, gene flow among divergent populations tends to erode genetic differentiation between them [75]. The degree of genetic differentiation between populations can thus provide a measure of the extent over which dispersal and gene flow are occurring among *B. tabaci* populations in a landscape. The fixation index (F_ST_) [76], is a commonly used measure of genetic differentiation between populations, ranging from 0 (no differentiation) to 1 (complete differentiation). Of the 15 studies reporting values of F_ST_ among populations within cryptic species, eight (53%) include ranges of F_ST_ that bound zero [17,41,54,58,61,65,66,67], though upper estimates of F_ST_ among studies sometimes exceed 0.5 [59,64,65] (Figure 2, Table S2). Differences in the geographic extent of sampling largely explain differences in the range of F_ST_ among studies. Studies reporting relatively low genetic differentiation between populations typically cover small geographic areas within individual countries [16,17,54,56,61,66], whereas those with high or broad ranges of genetic differentiation generally compared populations spanning multiple islands [63], numerous sites distributed throughout individual countries [58,59], or sites spanning multiple countries/continents [64,67]. Analysis of Molecular Variance (AMOVA) is another common tool used for reporting levels of genetic differentiation, functionally partitioning levels of genetic variance into hierarchical levels including categories for among-individuals within a population and among-populations within a region. The relative levels of genetic variance ascribed to these two categories can provide a measure of the amount of genetic differentiation among populations. The median percentage of genetic variance among individuals within populations across ten studies reporting AMOVA results based on microsatellite datasets was 86% (Figure 2), and only two studies exhibited <70%, corroborating findings of low F_ST_ among populations, and supporting the occurrence of high levels of gene flow among populations within cryptic species.

### 3.2. Interpreting K = 2

**Figure 3:**
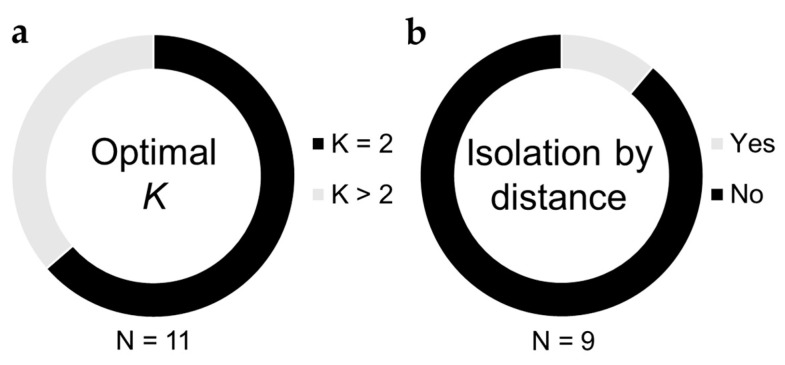
Summary of STRUCTURE and isolation by distance analyses. (**a**) Circle diagram depicting the proportion of studies reporting an optimal value of *K* = 2 versus *K* > 2. Values of *K* = 2 are generally interpreted to mean that there is little/no population structure distinguishing the sampled populations (**b**) Circle diagram depicting the proportion of studies reporting evidence of isolation by distance, a phenomenon wherein genetic differentiation (represented by F_ST_/(1-F_ST_)) increases with increasing geographic distance between populations.

Another often used measure of genetic differentiation among populations is the Bayesian clustering analysis implemented in the software STRUCTURE [77]. The optimal number of genetic clusters, *K*, is inferred using several heuristics, and proportions of ancestry coefficients among clusters for each individual are assessed for evidence of admixture or isolation among populations. A challenge that is common to all applications of STRUCTURE appears to be at play among *B. tabaci* studies as well, termed the “*K* = 2 conundrum” [78]. An optimal value of *K* = 2 was reported in 64% of studies (7 out of 11) applying STRUCTURE to the study of *B. tabaci* genetic differentiation [16,54,57,58,59,60,63] (Figure 3, Table S1). Because the possibility of *K* = 1 is not evaluated by STRUCTURE, an optimal value of *K* = 2 can sometimes imply the near-complete absence of genetic differentiation among populations, especially when ancestry proportions for the two genetic clusters are evenly mixed among most individuals [78]. Four of 11 studies reported optimal values of *K* > 2 (Figure 3, Table S1), but interpretation seems to be complicated by the inclusion of multiple cryptic species in the same analysis [55,69,71]. STRUCTURE results are therefore in agreement with F_ST_- and AMOVA-based analyses in suggesting that gene flow among populations can be quite high over relatively large spatial extents.

### 3.3. Limited Evidence of Isolation by Distance

When migration among populations decreases as a function of increasing distance between them, a pattern of isolation by distance can arise [79], which is often quantified by comparing levels of genetic differentiation (F_ST_/1-F_ST_) with the geographic distance between pairs of populations. An absence of evidence of isolation by distance can follow from frequent long-distance migration events erasing any association between genetic differentiation and geography. Of nine studies testing for evidence of isolation by distance [13,17,54,56,57,58,60,67,68], only one detected a significant association between genetic differentiation and geographic distance among populations within a region [58] (Figure 3, Table S1). Notably, isolation by distance was not observed over spatial extents as wide as the Mediterranean Basin, though most studies spanned regions within individual countries (Columbia, France, Greece, Réunion, Taiwan).

The one case where a significant pattern of isolation by distance was found [58] seems to have arisen from a combination of three otherwise non-unique factors. First, sampling occurred in greenhouses that were known to regularly receive plant materials from different sources each year [58]. While anthropogenic movement of *B. tabaci* via plant trade would otherwise be expected to erase any pattern of isolation by distance, as evident in [13,17,57,67], what, in Park et al. [58], essentially amounts to multiple introductions from separate sources each year, could produce a pattern of isolation by distance, especially if greenhouses tend to obtain seedlings from closer rather than more distant nurseries. Second, the spatial coverage of the study region (South Korea) was relatively broad compared to other studies that sampled from a single country [13,17,56,57,60], a factor that we earlier showed also enables detection of a broader range of genetic differentiation (F_ST_) among populations. This likely offered greater statistical power for subsequent tests of isolation by distance. Lastly, the density of sampling at each site was quite high (up to 40 individuals per site, while others usually sampled ~20 per site), again lending itself to greater resolution of any genetic differences among populations. So, while isolation by distance may appear rare in *B. tabaci*, we suggest that sampling more densely among sites that are well-distributed across a region (not too narrow geographically, but also not too broad) might reveal more cases where gene flow is geographically restricted among populations.

### 3.4. Pervasive Heterozygote Deficits

**Figure 4:**
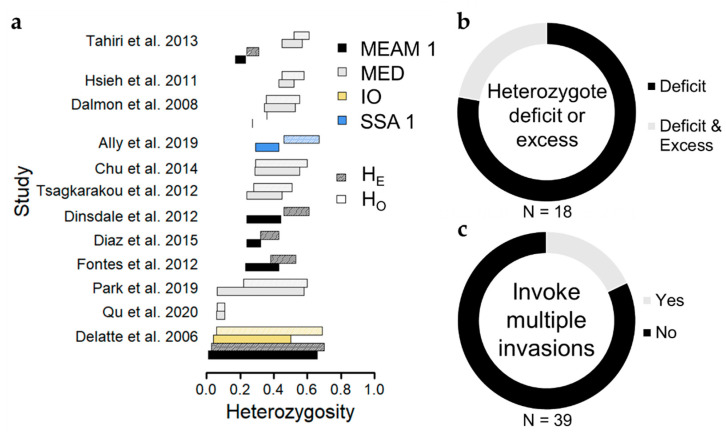
Summary of genetic diversity among populations within cryptic species among studies. (**a**) Barplots depicting the range of observed and expected heterozygosity for each cryptic species among studies [13,16,17,54,56,57,58,60,61,65,66,70]. “MEAM 1” stands for Middle East-Asia Minor 1; “MED” stands for Mediterranean; “IO” stands for Indian Ocean; “SSA 1” stands for Sub-Saharan Africa 1. (**b**) Circle diagram depicting the proportion of studies reporting heterozygote deficits (regardless of whether this was formally tested with a statistical model) among most if not all populations. (**c**) Circle diagram depicting the proportion of studies that interpreted patterns of genetic variation as evidence of multiple invasions having occurred in the study region.

A common theme throughout microsatellite-based studies reporting on genetic diversity among *B. tabaci* populations is the observation of widespread heterozygote deficits (Figure 4), which occur when observed heterozygosity is lower than expected based on Hardy–Weinberg Equilibrium assumptions, and which can be quantified using the inbreeding coefficient F_IS_ (F_IS_ > 0 indicates heterozygote deficit). Of eighteen studies reporting population estimates of observed and expected heterozygosity, 14 (78%) demonstrated heterozygote deficits in most populations [13,16,54,56,57,60,61,63,64,65,66,67,68,70], and 4 (22%) reported instances of heterozygote deficit alongside heterozygote excess [17,55,58,59], wherein observed heterozygosity exceeds expectations under Hardy–Weinberg Equilibrium (Figure 4, Table S1). Heterozygote excess in haplodiploid species, such as *B. tabaci*, can result from large differences in allele frequencies between sexes [80], but is often considered a signature of a recent population bottleneck (i.e., a dramatic reduction in population size) [81]. While some sampled populations appear to have undergone a population bottleneck, perhaps due to founder events typical of invasive populations or strong population suppression from insecticides [13,55,57], the majority of populations instead exhibit some degree of heterozygote deficit.

What is the cause of such pervasive heterozygote deficits and what can they tell us about the extent of gene flow among populations? One cause of heterozygote deficits could be genotyping error, a common challenge among microsatellite datasets [82], wherein allele size or primer binding variation leads to allele dropout (‘null alleles’) and misclassification of homozygous genotypes when they are truly heterozygous genotypes [59,64]. However, studies specifically account for genotyping error, and null alleles would not be expected to permeate every *B. tabaci* microsatellite dataset. One interpretation of heterozygote deficits is that a population is undergoing substantial inbreeding, a phenomenon where heterozygosity erodes due to frequent mating between closely related individuals [83]. This might follow from an absence of gene flow among divergent populations, suggesting that localized *B. tabaci* movements over the landscape may predominate, but could also arise if sampling schemes result in high rates of sibling sampling. Additional evidence of inbreeding in microsatellite datasets is strong linkage disequilibrium among loci, wherein allele frequencies among loci are correlated because they share a very recent common ancestor and are still being inherited together. Importantly, though, linkage disequilibrium across loci was rare among *B. tabaci* microsatellite studies. Without further evidence from genome-wide data (such as long homozygosity tracts), it is difficult to conclude that the observed heterozygote deficits stem from inbreeding. Lastly, consequences of *Wolbachia* infection have been proposed to explain heterozygote deficits, whether due to sex-ratio distortions [68] or asymmetrical gene flow due to cytoplasmic-incompatibility [67]. *Wolbachia* infections can be quite common among MEAM1 and MED, but also extremely rare, depending on geography [51,57,84,85]. The potential for horizontal transmission via shared host plants, in addition to vertical transmission, suggests that *Wolbachia* infections will only grow in importance with time [86]. However, studies examining the effect of *Wolbachia* infection status on *B. tabaci* population structure have so far found no causal relationship [67,68].

Instead, we propose that pervasive heterozygote deficits arise due to population substructure (i.e., non-random mating) among individuals collected at a site, referred to as the Wahlund effect [87]. Population substructure might follow from host-associated differentiation [88], but studies so far do not report evidence of host-associated differentiation among *B. tabaci* populations within cryptic species (Saurabh et al. this issue, [39,57]). Instead, population substructure likely reflects sampling of individuals as they immigrate from distinct populations, implying that movement occurs frequently and potentially over large spatial extents, but that this does not always result in gene flow among populations. Along these lines, multiple studies interpret observations of genetic variation among *B. tabaci* populations as evidence for multiple invasions in their study extent (Figure 4) [17,27,41,55,56,59], and unique MED COI haplotypes can co-occur on the same plant [72]. It is possible that divergent lineages of invasive *B. tabaci* populations are widely dispersing throughout agricultural landscapes but have not admixed enough to erase any Wahlund effect.

### 3.5. Other Lines of Evidence

Three other lines of evidence provide some support for the idea that *B. tabaci* are connected over large spatial extents: temporal shifts in genetic cluster assignment, rapid spread of symbiont infections, and widespread distributions of insecticide resistance mutations. Invasive MED populations exhibited large shifts in genetic cluster assignment over a period of six years in China [65], as a single COI haplotype came to predominate throughout most agricultural regions [44]. In Australia, invasive MEAM 1 populations exhibited substantial genetic change (according to AMOVA applied among populations over time) among seasons within a single year [60]. In addition to temporal changes due to the spread of invasive *B. tabaci*, distributions of native populations can also exhibit broad shifts over time, with the viruliferous SSA 1 spreading throughout much of Sub-Saharan Africa over a period of 20 years [61]. Second, a *Rickettsia* infection spread to nearly 100% of *B. tabaci* in 6 years over an area spanning southern Arizona, USA [31]. Lastly, findings of common, widespread insecticide resistance mutations (associated with organophosphate and pyrethroid resistance) throughout the Mediterranean Basin suggest that frequent migration is facilitating spread of advantageous alleles [67], though selection on standing genetic variation [89] within whitefly’s ancestral range cannot be ruled out. So, all of these lines of evidence support the conclusions drawn from F_ST_, AMOVA, and STUCTURE analyses that there is potential for ongoing gene flow among *B. tabaci* populations over broad spatial extents.

## 4. Single Nucleotide Polymorphisms

Only four studies have so far used single nucleotide polymorphisms (SNPs) to examine *B. tabaci* population genetics, and none were designed to address questions about gene flow among populations on small spatial and temporal scales [15,70,71,90]. Wosula et al. [71] sampled *B. tabaci* from cassava (*Manihot esculenta*) in 8 countries in Sub-Saharan Africa, and found relatively high levels of genetic differentiation (minimum F_ST_ = 0.11, *K* = 4) according to 7453 SNPs. However, analyses of population structure were complicated by the inclusion of multiple *B. tabaci* cryptic species. Chen et al. [69] obtained similar results after expanding sampling to 18 Sub-Saharan African countries and 63,770 SNPs. Elfekih et al. [15] genotyped MEAM 1, MED, and IO (“Indian Ocean”) whiteflies from 20 sites on six continents at 38,041 SNPs, but analyses of population structure were focused on revealing invasion routes among countries rather than ongoing movement patterns over landscapes. Qu et al. [70] found values of F_ST_ among MED populations between 0.078–0.346, but here comparisons were made among populations from different continents. While these studies were well designed for characterizing the species composition of economically important *B. tabaci* communities and genetic differences at continental and global scales, there remains a need for studies that leverage genome-wide datasets to address questions about the importance of local movements for *B. tabaci* ecology and evolution. Such studies in other insect systems have revealed colonization of crop fields from specific non-crop host plant reservoirs [4], identified sibling pairs separated by distances > 3 km and uncovered previously hidden patterns of isolation by distance [91], and shown how the spatial configuration of crop fields can impede or enhance gene flow among pest populations in certain environments [5,92].

## 5. Concluding Remarks and Future Directions

What is the spatial extent of a *B. tabaci* population? While our synthesis of the current *B. tabaci* population genetics literature cannot provide a quantitative answer, it does suggest that there is potential for frequent and widespread gene flow on the scale of an agricultural region. The limited resolution provided by small numbers of genetic markers in most studies, combined with the recent invasion history of many economic *B. tabaci* populations cast doubt on this interpretation and complicate the task of linking population genetic patterns with ecological and evolutionary processes. Low genetic differentiation and pervasive heterozygote deficits among populations could be a consequence of recent introduction of a small number of founding individuals, which would obscure any ability to detect evidence of geographic isolation or widespread gene flow among populations based on patterns of genetic differentiation. Moving forward, higher-resolution genetic data, like that offered by genome-wide single nucleotide polymorphism (SNP) datasets, combined with systematic sampling over landscapes and over time will play an important role in distinguishing whether populations are primarily local in nature or connected over wide spatial extents. Studies of *B. tabaci* population genetics are beginning to make use of SNP datasets [15,70,71,90], but have not yet been designed to address questions about dispersal and gene flow among populations in agricultural landscapes at local scales. As the time since invasion of most MEAM 1 and MED populations continues to increase, such studies may begin to reveal previously unknown connections among commodities and landscapes or delineate geographically isolated groups of economic *B. tabaci* populations.

We suggest three questions to guide future research along these lines. First, what are the genetic consequences of *B. tabaci* migration among cropping systems? Sampling whiteflies in a region over time as they first arrive and depart from emerging and senescing crops, respectively, could reveal important ongoing constraints on *B. tabaci* genetic variation that could potentially be modulated by crop diversity in landscapes. Second, what can whole-genome data reveal about the spatial extent of gene flow? The relative ease with which whole-genome data can now be generated for non-model systems opens the door for a powerful set of tools to be applied to the study of *B. tabaci* ecology and evolution. Pertinent to *B. tabaci* gene flow, genome-wide patterns of linkage disequilibrium can be used to make precise inferences about the spatial extent and timing of gene flow among populations [93], and haplodiploidy makes *B. tabaci* particularly amenable to this kind of analysis (because haploid male genomes contain perfect linkage information, circumventing the need for carefully constructed pedigrees). Lastly, is anything impeding movement of *B. tabaci* among crops? Landscape genetics approaches, which examine associations between genetic variation and environmental variables [94], provide a useful framework with which to approach this question where evidence for genetic differentiation among populations is found [95], and are still rarely applied in agricultural pest systems [5,7,96]. Discerning whether *B. tabaci* populations should be considered on local versus regional scales is important for designing management schemes of all kinds, including, but not limited to, insecticide resistance management strategies, which could benefit from coordinated efforts to rotate insecticide modes of action across commodities and regions where *B. tabaci* population linkages are evident.

## Supplementary Materials

The following are available online at https://www.mdpi.com/2075-4450/11/11/813/s1, Table S1: Summary of select attributes of reviewed research articles, Table S2: Summary of genetic diversity and differentiation among cryptic species and studies.
